# Correction to: Forkhead box (FOX) G1 promotes hepatocellular carcinoma epithelial-Mesenchymal transition by activating Wnt signal through forming T-cell factor-4/Betacatenin/FOXG1 complex

**DOI:** 10.1186/s13046-021-01900-2

**Published:** 2021-03-17

**Authors:** Xingrong Zheng, Jiaxin Lin, Hewei Wu, Zhishuo Mo, Yunwen Lian, Peipei Wang, Zhaoxia Hu, Zhiliang Gao, Liang Peng, Chan Xie

**Affiliations:** 1 Department of Infectious Diseases, the Third Affiliated Hospital of Sun Yat-sen University, 600# Tianhe Road, Guangzhou, 510630 Guangdong Province China; 2 Key Laboratory of Tropical Disease Control, Ministry of Education, Sun Yat-sen University, Guangzhou, 510630 Guangdong Province China; 3 Guangdong Provincial Key Laboratory of Liver Disease, Guangzhou, China

**Correction to: J Exp Clin Cancer Res 38, 475 (2019)**

**https://doi.org/10.1186/s13046-019-1433-3**

Following publication of the original article [1], the authors identified some minor errors in image-typesetting in Fig. 2; specifically in Fig. 2e.

The corrected figure is given below. The correction does not have any effect on the results or conclusions of the paper.

**Fig. 2:**
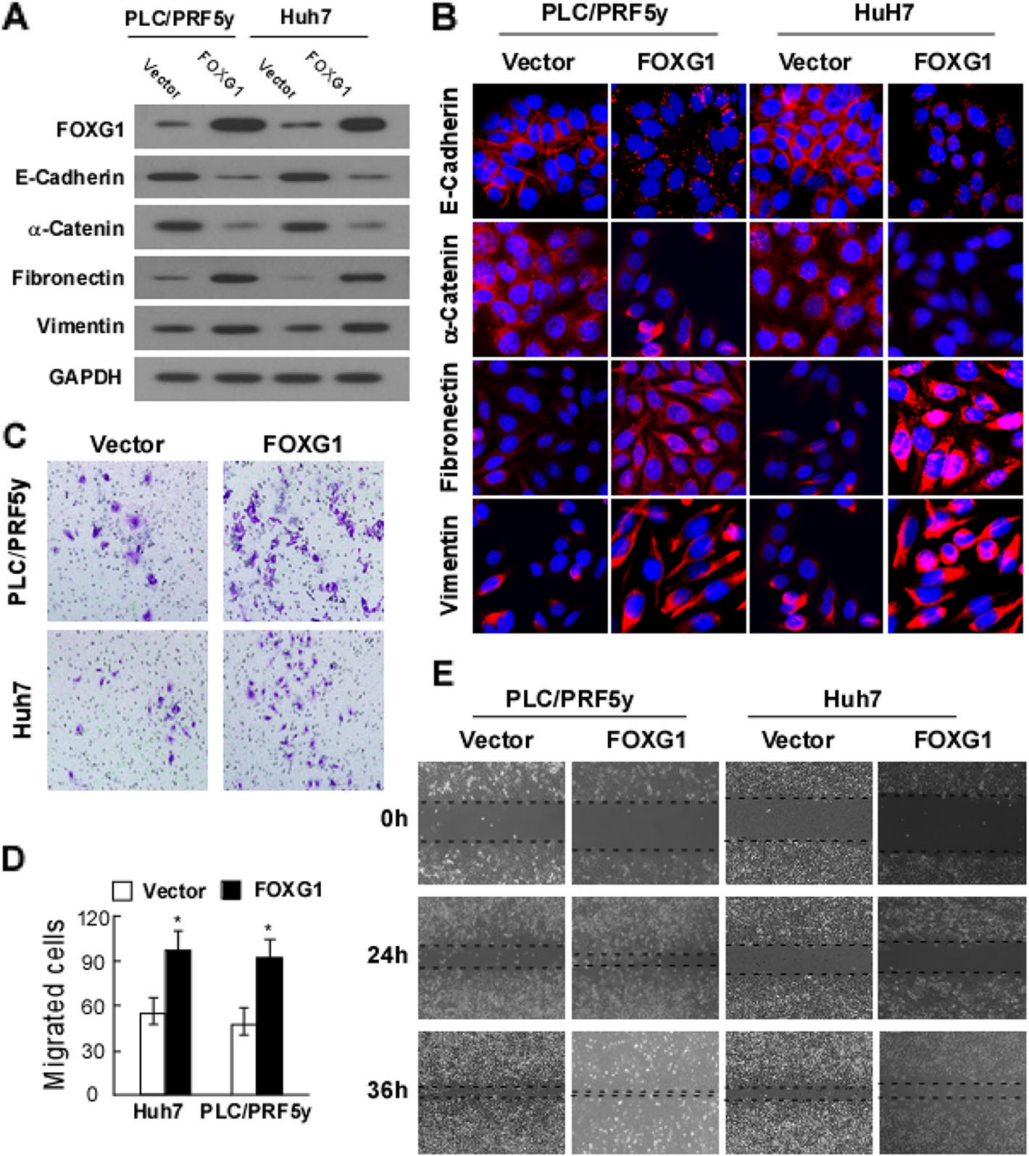
Overexpression of FOXG1 promotes cell mobility and invasion by inducing epithelial-mesenchymal transition (EMT). **a** Western blotting analysis and **b** Immunofluorescence analysis of expression of epithelial cell markers (E-cadherin and α-catenin) and mesenchymal cell markers (vimentin and fibronectin) in indicated cells transfected with FOXG1 expression vector or control vector. Nuclei counterstained with DAPI. GAPDH was used as a loading control. **c** Representative migrating images of the indicated Huh7 and PLC/PRF5y cells on uncoated Transwell devices in five random fields. **d** Quantification of the invading cells of the indicated Huh7 and PLC/PRF5y cells on Matrigel-coated Transwell devices in five random fields. Values represent mean ± SD. *P < 0.05. **e** Representative micrographs of wound healing assay of the indicated Huh7 and PLC/ PRF5y cells. Wound closures were photographed at 0, 24, and 36 h after wounding. All experiments were repeated at least three times with similar results
